# Supplementary material for: Structural equation model of psychological constructs of transtheoretical model, motives for physical activity, and amount of physical activity among people with type 2 diabetes mellitus in Malaysia
Source: PLoS One. 2022 Mar 31;17(3):e0266104. doi: 10.1371/journal.pone.0266104 (PMC8970478; doi:10.1371/journal.pone.0266104)
Supplement: S1 Data — (PDF) [file pone.0266104.s001.pdf]

SOC	Cognitive	Behaviour	Pros	Cons	SE	Competition	Appearance	OExpectat	Affiliation
2	62	51	21	19	38	8	11	13	7
3	52	48	18	15	49	13	13	15	14
3	52	42	15	24	39	18	16	12	15
2	50	31	13	25	18	16	20	16	18
6	55	59	20	15	54	18	17	14	18
3	54	41	19	24	63	10	9	13	10
1	40	20	10	16	26	11	20	14	15
3	51	28	14	15	34	13	15	13	15
3	45	24	9	21	44	16	20	16	16
6	48	31	15	17	26	16	20	13	20
1	44	20	11	15	23	12	19	14	15
3	56	49	18	12	42	18	19	12	19
5	56	56	19	11	42	20	20	12	18
3	56	53	18	14	18	19	17	13	18
3	32	36	20	12	36	19	19	13	16
5	54	72	24	5	25	25	23	15	25
5	69	60	24	25	65	17	19	12	15
4	48	53	20	10	47	20	19	17	19
3	54	45	18	24	45	15	16	18	19
3	52	34	18	20	29	15	20	15	16
3	56	59	20	10	53	20	20	17	20
2	40	27	13	13	25	11	16	12	12
5	53	46	15	16	56	13	13	15	14
3	55	52	19	18	32	18	21	15	18
3	42	58	20	20	53	14	18	13	14
2	44	45	15	18	53	16	15	16	15
3	53	59	23	22	39	21	25	19	23
3	61	53	20	17	53	22	24	19	19
3	52	37	21	23	64	13	12	14	15
2	51	41	13	14	30	15	19	14	17
3	54	45	19	18	54	14	18	14	18
3	53	33	18	23	54	14	17	13	16
3	47	38	24	15	74	16	14	15	16
6	40	27	13	16	40	7	15	13	7
5	55	60	21	24	30	8	15	16	10
2	55	37	18	16	30	19	18	17	17
2	60	60	19	23	53	17	21	17	19
3	63	68	23	16	42	22	25	22	15
5	64	61	21	14	64	24	25	19	24
2	39	27	12	23	37	12	12	14	10
1	57	69	15	16	48	20	20	16	20
3	53	43	18	19	33	14	20	12	17
3	44	42	17	15	51	17	15	14	16
3	51	42	19	25	87	10	10	14	10
5	59	61	25	16	57	25	25	17	21
3	56	50	20	22	35	11	18	16	15
2	54	42	15	17	49	12	14	13	13
3	55	47	22	23	47	13	24	14	16
5	50	41	16	22	41	8	8	13	8

3	55	38	15	23	24	15	17	13	18
3	28	19	13	24	19	8	7	16	9
3	56	50	15	25	39	7	5	12	9
6	65	65	25	16	56	20	25	16	23
1	40	26	12	14	23	12	15	13	14
3	56	48	19	11	23	13	18	13	18
1	29	17	9	13	18	10	15	13	11
5	43	32	18	17	23	15	20	15	15
3	63	43	20	15	42	18	20	13	19
1	53	38	21	18	32	15	19	13	17
2	50	32	15	22	56	10	18	10	18
3	51	39	15	17	34	11	14	14	15
3	18	36	14	22	35	15	16	12	15
3	57	45	21	18	42	11	20	14	14
3	38	45	17	23	33	15	21	13	13
3	43	41	21	18	31	18	19	15	15
2	43	32	18	21	37	10	13	12	17
2	39	28	10	22	21	16	15	11	15
1	33	27	16	24	18	12	8	15	11
1	50	45	25	15	26	9	16	15	21
5	57	48	25	19	57	12	23	15	18
3	34	18	15	20	18	13	12	18	12
2	50	57	25	14	25	7	20	17	14
3	31	39	22	20	50	14	14	13	11
1	32	19	12	18	18	7	6	12	5
2	43	34	13	18	31	10	14	13	16
2	43	34	13	18	31	10	14	13	16
3	48	33	15	23	35	10	14	13	15
3	48	33	15	23	40	10	14	13	15
3	48	33	15	23	35	10	14	13	15
3	48	33	15	23	35	10	14	13	15
2	33	43	14	24	36	15	16	13	14
3	42	42	16	23	36	15	17	16	16
3	43	37	13	21	38	13	13	10	12
3	48	33	15	23	35	15	15	9	15
3	43	46	16	17	37	15	14	15	14
3	48	33	15	23	32	10	15	13	15
2	35	39	13	21	31	12	14	13	11
5	35	58	16	25	34	10	10	15	25
1	40	33	14	22	24	14	16	11	14
2	38	45	14	18	47	15	17	13	15
2	45	44	15	22	28	13	15	11	17
2	43	37	14	22	31	13	16	11	15
3	40	35	15	22	37	14	15	10	16
2	42	33	14	22	28	12	14	11	15
2	39	35	15	21	34	15	17	10	17
2	39	34	15	22	26	10	15	9	13
1	40	35	15	22	38	12	12	13	11
2	35	41	14	22	37	16	15	13	13
1	41	38	14	22	28	12	13	12	12

1	40	36	14	23	33	11	15	12	13
1	43	32	13	22	28	13	11	10	15
1	40	38	14	22	33	13	14	12	12
2	41	35	13	22	31	10	14	10	12
1	41	33	14	22	34	14	14	12	14
2	41	43	14	16	32	13	11	13	12
1	43	34	13	23	31	14	11	15	13
3	41	37	14	21	31	14	15	11	14
2	46	36	14	22	36	15	15	11	13
2	43	34	14	22	28	11	13	11	15
3	42	41	14	22	36	12	14	12	14
1	44	33	13	22	25	10	13	10	14
3	45	41	15	22	36	12	14	12	15
3	44	38	14	22	41	15	14	10	16
3	45	40	15	20	28	11	14	11	15
3	45	36	14	22	28	12	12	10	15
2	39	35	14	22	22	14	15	11	13
3	46	35	14	22	30	11	16	12	12
2	43	38	15	22	27	12	11	10	16
3	42	35	14	20	36	14	15	11	12
1	39	34	16	22	24	12	14	10	15
1	40	40	13	22	25	12	13	9	14
1	42	37	15	22	34	13	12	9	14
1	41	44	14	22	34	12	10	13	11
2	45	37	12	21	34	12	15	12	13
3	47	46	17	22	37	14	13	11	13
3	40	44	13	21	35	16	16	12	16
3	41	40	16	21	38	14	14	11	13
2	41	40	14	22	28	15	15	13	15
2	41	37	14	22	31	13	14	12	14
2	43	34	13	18	30	10	14	13	16
2	43	34	13	18	32	10	14	13	16
2	43	34	13	18	32	10	14	13	16
2	43	34	13	18	32	10	14	13	16
2	43	34	13	18	32	10	14	13	16
2	43	34	13	18	32	10	14	13	16
1	38	32	13	22	22	11	13	12	12
3	43	38	14	22	31	11	15	11	15
3	45	41	14	22	49	12	15	10	16
1	18	21	7	16	18	6	6	10	7
3	45	40	14	22	27	13	14	10	14
2	41	36	15	22	33	11	12	12	13
2	37	34	13	18	30	10	14	13	15
2	46	44	15	15	44	15	15	13	13
1	44	38	11	22	31	10	9	12	11
3	44	38	14	22	30	14	17	10	15
3	39	33	14	22	24	12	17	10	15
1	40	30	14	22	22	12	12	11	14
1	15	15	5	25	18	5	5	10	5
1	25	26	12	18	26	11	7	9	8

1	23	22	12	20	18	9	8	9	8
1	40	49	20	13	18	14	15	9	19
2	32	28	20	14	18	16	20	12	20
1	59	39	20	21	33	16	21	10	20
1	36	25	20	17	20	5	20	11	12
1	15	15	5	22	18	5	5	9	5
1	26	22	16	23	23	7	5	17	8
1	27	27	15	17	22	12	6	13	6
1	44	22	19	25	19	9	5	16	5
1	15	15	5	25	18	5	5	9	5
2	49	33	13	21	18	18	22	18	17
1	50	30	23	23	18	8	18	13	5
1	55	23	20	22	18	7	14	8	20
3	37	30	12	20	18	17	17	7	15
1	51	38	14	17	36	10	19	15	18
1	24	26	8	25	18	14	12	8	11
1	48	30	23	5	18	17	16	12	17
1	36	22	10	24	18	5	8	12	6
3	58	41	20	19	26	12	24	9	23
1	21	16	10	16	18	15	5	11	5
1	44	25	18	19	20	5	6	12	6
3	30	22	15	22	18	8	7	15	6
1	20	15	10	20	18	6	5	14	5
1	19	15	8	20	18	6	5	13	5
3	30	20	14	18	21	11	7	12	8
1	22	16	6	23	18	7	5	12	5
1	21	15	9	22	18	6	6	16	5
1	21	17	9	25	18	6	6	10	5
1	19	15	7	25	18	6	5	14	5
2	34	29	18	21	23	9	9	16	9
2	39	33	18	22	21	14	8	16	16
1	31	20	10	18	18	8	8	17	8
3	41	32	20	24	37	12	11	14	13
5	30	25	15	21	18	12	10	17	10
1	34	15	9	15	18	5	5	11	7
1	35	23	11	14	20	8	5	13	9
1	33	24	19	25	18	8	5	16	6
3	29	18	14	23	18	11	5	17	5
3	31	22	14	19	18	10	11	15	8
3	59	53	20	19	63	19	20	17	20
3	49	43	19	25	37	13	20	16	15
6	69	45	25	7	72	20	20	15	20
5	70	46	20	24	49	16	19	15	19
5	62	62	23	18	67	20	21	13	22
3	60	56	18	23	59	19	22	15	17
3	56	40	19	14	47	12	15	17	16
5	53	56	17	19	54	16	20	20	14
3	43	32	18	21	37	10	13	12	17
3	43	32	18	21	37	10	13	12	17
1	32	23	13	19	18	6	5	17	7

1	33	32	14	19	22	5	5	16	10
4	30	33	22	21	50	9	16	16	8
3	24	15	8	25	18	6	5	11	5
5	41	58	23	18	45	15	16	13	22
6	31	25	16	22	18	12	12	17	8
3	40	38	19	23	22	12	10	14	10
1	22	16	8	25	18	6	6	13	5
3	45	41	14	23	34	15	13	11	14
3	42	38	15	23	28	12	13	11	14
1	45	38	13	21	23	12	13	11	15
3	43	39	14	22	27	11	13	9	14
3	42	38	14	22	28	13	12	13	15
2	41	43	13	22	31	12	14	11	14
1	41	39	11	22	24	10	14	9	12
3	42	36	15	22	35	14	14	9	14
2	39	29	14	22	32	12	14	10	15
3	45	39	14	22	37	15	15	13	13
2	37	33	10	22	26	13	13	11	13
2	46	41	15	22	29	13	14	10	15
3	43	39	15	23	30	11	16	9	15
3	44	39	13	22	27	13	14	10	14
3	46	40	15	23	33	15	13	12	14
3	47	40	13	23	31	13	12	11	15
3	44	42	15	22	29	14	14	12	14
2	42	38	12	22	25	12	15	9	14
2	43	37	12	22	25	12	15	10	14
3	45	39	13	22	29	11	14	11	14
3	42	44	14	22	34	13	15	11	15
3	42	41	14	22	31	14	15	10	14
2	39	30	14	22	25	12	12	10	14
3	39	40	14	22	34	11	15	11	14
1	36	34	11	23	26	11	13	10	12
2	42	33	12	22	25	14	13	10	14
1	38	36	11	22	25	13	12	12	13
3	41	41	15	22	37	13	14	9	16
3	42	36	15	22	32	14	12	13	14
3	44	39	15	22	33	14	15	12	13
1	37	33	11	22	25	10	11	10	14
1	37	34	12	23	25	11	12	9	13
2	39	32	15	20	24	12	15	10	14
3	44	41	16	22	41	13	13	10	14
3	45	38	15	22	31	14	14	13	15
3	46	38	13	22	33	15	14	11	14
3	44	37	13	22	40	14	16	10	14
2	41	36	13	21	28	14	15	14	14
3	44	39	12	22	30	15	13	12	13
2	40	35	13	22	29	14	12	11	15
3	45	41	12	22	34	11	13	10	14
2	41	34	12	22	25	14	15	11	13
3	45	40	15	22	40	16	15	11	15

3	43	40	15	22	36	13	14	12	14
1	40	33	11	23	29	12	13	10	13
2	37	35	13	22	28	12	14	11	15
3	40	30	15	22	29	15	15	12	15
1	40	31	11	22	25	11	12	12	12
1	39	36	15	22	25	13	14	11	14
3	39	39	15	22	29	13	16	10	11
1	36	27	12	22	29	11	14	12	15
1	36	27	12	22	29	11	14	12	15
1	36	29	12	22	29	11	14	12	15
1	36	27	12	22	29	11	14	12	15
1	36	27	12	22	29	11	14	12	15
1	36	27	12	22	29	11	14	12	15
1	36	27	12	22	29	11	14	12	15
1	36	27	12	22	29	11	14	12	15
1	36	27	12	22	29	11	14	12	15
1	36	28	12	22	29	11	14	12	15
1	32	27	12	22	29	11	14	12	15
1	32	27	12	22	29	11	14	12	15
1	32	27	12	22	29	11	14	12	15
1	32	27	12	22	29	11	14	12	15
1	27	35	13	14	38	10	10	12	9
1	37	27	11	20	33	11	14	12	15
1	37	27	11	20	33	11	14	12	15
2	44	42	15	22	36	13	10	12	12
3	40	37	14	22	35	14	14	12	13
1	38	34	14	22	36	14	14	14	12
3	39	31	15	22	33	15	15	14	14
1	32	38	14	22	27	14	11	14	12
2	39	40	14	23	30	13	12	12	12
3	38	39	14	23	32	14	13	14	12
1	41	37	11	21	37	14	13	12	10
3	42	33	13	22	36	12	12	11	14
3	34	35	14	22	39	12	12	13	12
1	34	39	12	23	32	13	11	11	12
1	37	38	13	23	32	11	12	10	9
1	37	39	14	23	30	12	10	11	13
1	41	35	14	22	34	15	11	13	12
3	43	37	13	22	35	13	9	13	11
2	39	33	13	19	32	14	10	14	11
3	42	39	13	22	31	12	8	11	13
3	38	38	13	20	36	12	10	11	12
2	33	37	12	23	31	13	13	14	12
3	35	37	11	19	37	13	11	13	10
2	33	39	13	17	28	13	11	11	13
1	27	36	12	20	32	12	12	12	13
3	30	38	12	19	32	12	11	14	10
3	33	44	12	18	32	13	10	12	11
2	33	37	12	18	28	11	10	11	12
2	31	40	11	19	33	12	12	12	11

3	35	45	12	18	40	14	11	12	14
3	32	38	12	18	38	11	11	11	8
2	32	36	9	18	40	12	11	12	12
3	40	42	13	19	49	11	11	13	12
1	24	37	10	20	30	10	13	8	11
2	36	38	12	18	32	11	10	10	12
3	35	42	14	18	39	13	12	11	9
2	56	48	20	19	54	9	13	14	13
1	39	33	12	20	19	11	10	14	15
3	49	35	17	19	31	13	14	14	15
3	43	42	16	18	29	11	15	12	15
2	41	42	15	18	30	11	15	12	15
1	42	33	14	15	28	9	14	12	15
2	43	28	12	15	24	11	15	12	13
3	36	31	12	15	28	9	15	13	15
2	36	34	15	15	29	8	14	13	15
1	38	34	15	15	27	9	13	14	15
1	37	33	15	15	27	9	15	13	15
1	35	37	14	20	31	12	12	13	13
3	31	37	17	14	55	18	20	15	19
3	49	44	20	16	32	16	19	16	17
1	18	23	10	17	23	13	12	12	12
3	41	37	12	17	27	16	14	13	15
5	42	43	12	15	46	13	13	13	13
3	32	22	15	15	56	15	16	14	14
3	29	37	13	17	37	16	19	11	18
6	46	35	20	15	26	13	20	12	15
3	24	33	12	18	24	16	19	11	18
1	21	23	10	22	33	13	11	10	13
3	39	36	14	15	37	20	18	12	19
5	55	30	15	15	22	15	15	12	15
3	51	42	15	18	20	16	15	14	15

PhysicCond	PsychCond	Mastery	Enjoyment	AmountPA
25	15	12	13	0
14	14	14	11	0
20	18	18	19	0
20	18	20	18	0
19	19	20	19	586.5
17	14	10	12	396
20	19	13	18	0
14	13	18	14	570
20	18	16	18	720
20	19	16	19	930
18	16	14	16	165
20	15	20	19	1902
22	20	20	20	2373
20	16	18	15	725
17	17	18	16	1080
25	23	25	24	1853
22	19	20	21	4293
20	20	18	19	558
24	22	18	21	1732.5
20	19	18	19	1071
20	20	20	20	831
18	15	15	14	311
19	16	17	17	1022
20	19	20	20	2151
19	17	17	16	0
19	15	16	16	834
25	23	24	25	339
25	22	23	22	1200
23	17	15	16	285
19	17	19	18	0
24	18	19	19	60
21	20	19	16	1071
23	16	16	18	730
13	15	13	11	407.5
21	16	16	15	622
20	19	20	15	0
23	21	23	22	1257
25	20	24	24	1210
25	24	23	24	716
13	12	10	14	0
20	20	20	20	0
24	18	18	18	320
16	15	17	16	66
18	10	12	10	160
25	25	25	25	1262
20	17	16	16	0
15	15	13	13	0
22	18	19	15	972
20	20	14	19	2142

19	15	16	17	600
11	9	7	6	459
11	15	8	16	906.5
25	25	25	25	678
15	14	13	15	0
20	17	17	16	297
15	14	12	14	0
20	19	19	20	636
20	19	19	18	615
20	19	19	19	631
20	18	18	18	0
19	19	14	15	367.5
20	16	18	16	160
19	20	18	17	948.5
24	19	14	20	271
20	19	19	20	786.5
20	20	14	15	99
17	16	16	17	0
17	16	7	20	0
25	25	13	21	840
25	24	17	24	702
19	13	11	18	939
22	21	10	16	579
25	22	18	22	915
16	10	6	7	360
19	17	14	15	99
19	17	14	15	99
20	19	15	15	106
20	19	15	15	169.5
20	19	15	15	126
20	19	15	15	193
16	13	17	15	66
15	14	18	16	129.5
9	9	12	9	113
17	18	14	16	113
15	12	16	15	146
20	18	15	15	233
12	11	13	11	49.5
25	25	25	25	1866
13	12	14	13	0
16	14	16	14	66
16	13	15	14	0
17	13	15	12	0
17	12	13	13	138
16	13	13	16	0
19	12	15	15	129.5
14	11	13	11	33
15	11	14	12	0
17	15	13	16	126
15	11	14	12	0

14	13	12	14	0
15	11	12	10	0
17	12	12	9	99
15	12	13	12	49.5
14	14	13	12	0
17	11	14	11	49.5
15	12	15	11	0
15	13	13	11	0
16	12	14	11	33
15	12	13	14	0
18	13	15	14	0
13	11	14	13	0
17	14	17	14	129.5
17	13	13	14	120
15	13	13	14	93
16	12	12	15	139
14	13	14	13	0
16	13	17	15	129.5
17	11	16	15	80
16	12	12	16	126
13	12	13	14	0
15	11	14	12	0
15	13	13	16	0
14	10	12	10	0
16	14	15	13	0
17	13	15	16	153
17	13	15	16	129.5
18	12	13	13	139
15	14	16	16	0
16	11	13	11	0
19	17	14	15	146
19	17	14	15	188.5
19	17	14	15	179
19	17	14	15	33
19	17	14	15	99
19	17	14	15	99
15	11	12	11	0
17	13	16	14	120
18	13	13	15	0
6	8	5	7	0
18	13	15	16	33
15	11	13	12	73
18	18	14	15	66
18	13	15	17	106
15	10	10	9	0
16	13	15	15	120
15	12	14	13	33
14	13	13	13	0
5	5	5	5	0
9	7	9	8	932

6	5	7	8	0
16	15	16	18	0
20	20	13	14	0
20	22	19	24	0
20	17	9	15	0
5	5	5	5	0
19	9	8	9	0
19	13	5	10	906
19	18	15	10	3393
5	5	5	5	0
20	21	19	14	1386
25	23	6	15	0
20	17	8	12	0
18	18	17	15	0
20	17	14	20	219
14	13	12	11	0
17	19	19	20	0
9	8	6	7	0
21	22	14	23	1320
10	10	6	6	0
21	20	5	15	360
17	14	9	9	1828.5
12	8	5	5	0
9	5	7	5	0
16	14	9	8	328.5
12	7	6	5	0
14	9	5	5	0
8	7	7	7	0
11	9	5	7	0
20	15	11	17	0
23	19	12	19	120
18	15	9	11	720
24	19	10	15	5040
21	16	12	14	0
11	6	6	5	40
20	18	7	14	180
13	15	8	9	300
19	14	10	11	360
17	15	7	14	1828.5
20	18	20	20	360
20	20	11	18	0
20	20	20	20	231
21	20	17	20	1130
23	23	16	21	36.5
22	23	21	22	692
20	18	16	16	0
16	18	14	14	1198
20	20	14	15	99
20	20	14	15	99
20	17	8	11	60

21	19	8	14	1038
25	22	11	17	1186.5
10	5	8	6	0
25	20	14	21	791
20	16	10	13	360
22	15	13	17	504
9	7	6	5	0
18	12	14	15	80
16	13	15	13	109.5
13	12	15	13	0
18	12	14	14	80
18	13	15	14	33
14	12	13	13	0
14	11	13	13	0
17	13	13	15	139
15	13	13	14	0
17	12	11	15	66
16	12	13	14	0
14	14	14	13	49.5
18	14	16	13	99
17	13	13	14	80
17	13	15	15	60
18	13	16	13	153
17	13	14	15	113
14	12	14	14	33
16	13	15	12	0
17	14	15	15	49.5
18	14	15	16	73
17	14	14	14	33
16	13	12	12	33
18	14	15	14	93
14	13	12	14	0
15	13	13	12	0
14	11	13	12	0
17	13	16	14	66
16	12	15	14	148.5
19	13	14	15	33
14	13	13	14	0
14	12	13	12	0
13	12	14	14	0
17	13	18	15	66
18	12	16	15	80
17	13	14	14	0
18	15	17	16	80
16	12	14	14	0
14	11	15	14	0
15	13	14	16	33
16	13	13	14	148.5
14	11	14	12	0
18	15	17	15	160

17	13	14	14	169.5
15	12	11	14	33
16	12	15	14	0
17	12	14	14	93
14	11	12	13	0
15	11	12	14	0
15	12	13	14	60
20	15	15	15	0
20	15	15	15	33
20	15	15	15	0
20	15	15	15	0
20	15	15	15	0
20	15	15	15	0
20	15	15	15	99
20	15	15	15	33
20	15	15	15	66
20	15	15	15	148.5
20	15	15	15	33
20	15	15	15	66
20	15	15	15	49.5
20	15	15	15	0
10	10	13	9	0
20	15	15	15	33
20	15	15	15	0
14	11	15	11	0
15	14	13	12	33
16	12	14	13	0
15	13	13	13	129.5
13	9	14	11	0
13	8	13	9	49.5
15	11	15	12	126
16	11	13	13	0
13	12	12	10	148.5
14	11	14	11	99
15	12	13	10	66
14	9	8	10	33
14	11	11	11	0
15	10	14	11	66
12	11	12	11	153
15	10	10	9	33
13	12	11	11	153
12	10	12	12	129.5
13	11	14	12	33
14	9	11	9	126
16	11	12	10	33
14	11	13	12	0
11	10	12	9	99
12	9	12	12	148.5
12	11	13	12	66
12	12	12	12	66

16	10	13	12	129.5
14	12	10	12	126
12	13	13	12	33
10	10	11	13	106
13	11	10	8	0
12	7	12	7	33
13	10	11	10	129.5
15	16	16	14	318
20	17	14	15	231
15	15	15	16	160
15	15	15	13	358
15	16	15	16	198
16	15	15	15	99
15	15	14	13	99
15	15	15	14	219
16	16	15	15	212
15	15	14	16	198
15	15	15	15	148.5
14	14	12	14	66
19	18	18	17	565
20	18	16	15	66
11	12	12	10	0
13	14	14	15	186
13	12	13	13	645
19	17	18	17	379
18	19	17	16	219
21	20	18	18	148.5
18	18	16	17	0
15	14	13	14	0
16	18	21	20	405
16	15	15	15	594
16	15	15	15	0
