# Supplementary material for: Structural equation model of psychological constructs of transtheoretical model, motives for physical activity, and amount of physical activity among people with type 2 diabetes mellitus in Malaysia
Source: PLoS One. 2022 Mar 31;17(3):e0266104. doi: 10.1371/journal.pone.0266104 (PMC8970478; doi:10.1371/journal.pone.0266104)
Supplement: Attachment — Submitted filename: (F) suggestion.pdf [file pone.0266104.s002.pdf]

Title : Structural Equation Model of Psychological Constructs of Transtheoretical Model, Motives for Physical Activity, and Physical Activity Amount in People with Type 2 Diabetes Mellitus

Manuscript No : PONE-D-21-23408

---

Suggestion:

1. What is the meaning of “Psychological Constructs” representing in the present study?
2. Lack of rationale to support the model. SEM use to test the model not to develop model. Therefore, need citation/ theories/ model to support the indirect/ mediation variable/ structure of the model.
    a. Why TTM as mediator (even mediation not in the analysis but the present paper focus on indirect effect)?
    b. Kindly present and report direct effect when reported indirect effect.
    c. Kindly present the outcome of measurement model in brief.
    d. Three model reported and final Model 3 used according to the model fit values. However, which item(s) deleted or not fulfil the model requirement. Too brief and not clear, kindly enhance.
    e. Table 3 present the significant path, how about the non-significant path(s). The path deleted from model during model fit or during the measurement model analysis or during the structural model analysis ?
    f. How the value of indirect effect calculates? Where the significant value from?
3. What the age (years) of the participants? Kindly report in group (such as 20 to 30 years old etc) not mean.
4. Discussion too brief and more focus on reported previous studies. Discussion should align with the purpose of the study.
    a. What the overall model contribution? – 23 % refer to good or moderate etc ?
    b. What the contribution of indirect effect presented in this model outcome? What’s the value or impact of the findings?
5. No Implication and further research suggestion. Kindly revise and enhance the Discussion.
