# Supplementary material for: Structural equation model of psychological constructs of transtheoretical model, motives for physical activity, and amount of physical activity among people with type 2 diabetes mellitus in Malaysia
Source: PLoS One. 2022 Mar 31;17(3):e0266104. doi: 10.1371/journal.pone.0266104 (PMC8970478; doi:10.1371/journal.pone.0266104)
Supplement: Attachment — Submitted filename: Response to Reviewers.docx [file pone.0266104.s003.docx]

Dear Hoh Boon-Peng,

On behalf of my co-authors, I would like to thank you for your consideration of our manuscript, titled “Structural Equation Model of Psychological Constructs of Trantheoretical Model, Motives for Physical Activity, and Amount of Physical Activity among People with Type 2 Diabetes Mellitus in Malaysia”. We thank the Editor and the Reviewers for the careful consideration of our manuscript and for the largely favourable appraisal of this work. We also appreciate the Editor’s and Reviewers’ comments and think that the comments substantially helped us enhance the paper’s overall quality. As such, we highly appreciate the opportunity to revise and resubmit the paper. We hope that we have now adequately addressed the Editor’s and reviewer’ concerns and that you will now find our manuscript a useful addition to PLoS One. However, should there be any further issues requiring our attention, we would be grateful for a further opportunity to rectify these. Below, you will find our detailed responses to the comments.

**Editor’s comments and authors’ responses:**

**Journal staff:**

1. Please ensure that your manuscript meets PLOS ONE's style requirements, including those for file naming. The PLOS ONE style templates can be found at

https://journals.plos.org/plosone/s/file?id=wjVg/PLOSOne_formatting_sample_main_body.pdf and

<https://journals.plos.org/plosone/s/file?id=ba62/PLOSOne_formatting_sample_title_authors_affiliations.pdf>

Response: We have ensured that the manuscript meets PLOS ONE’s style requirement.

2. Please review your reference list to ensure that it is complete and correct. If you have cited papers that have been retracted, please include the rationale for doing so in the manuscript text, or remove these references and replace them with relevant current references. Any changes to the reference list should be mentioned in the rebuttal letter that accompanies your revised manuscript. If you need to cite a retracted article, indicate the article’s retracted status in the References list and also include a citation and full reference for the retraction notice.

Response: We have reviewed and ensured the reference list is complete and correct.

**Reviewer #1:**

1. What is the meaning of “Psychological Constructs” representing in the present study

Response: “Psychological Constructs” in the present study represent the psychological variables of the Transtheoretical Model which include, Processes of change, Decisional balance, and Exercise Self-efficacy. See line 78-82.

2. Lack of rationale to support the model. SEM use to test the model not to develop model. Therefore, need citation/theories/model to support the indirect/mediation variable/structure of the model

Response: We thank the reviewer for the suggestion. We added a citation in the last paragraph of Introduction section regarding rationale to support the model. See line 99-103.

a. Why TTM as mediator (even mediation not in the analysis but the present paper focus on indirect effect)?

Response: The indirect relationship is part of the SEM analysis. It is not the focus of the paper. But it provides additional information about the indirect relationship of motives for PA and psychological constructs on the amount of PA. If the indirect relationship is significant, it indicates that there is a significant mediator in the model.

b. Kindly present and report direct effect when reported indirect effect

Response: We thank the reviewer for the suggestion. Direct effects are reported in Table 3 (standardised regression coefficient). Thus, we do not repeat the direct effects results in Table 4.

c. Kindly present the outcome of measurement model in brief.

Response: We thank the reviewer for the suggestion. Outcome of measurement model presented in section “Measures and Materials” as we adopted the translated Malay version of all the scales from the previous validation studies (see line 124-183). Thus, the measurement model is not described in the present paper.

d. Three model reported and final Model 3 used according to the model fit values. However, which item(s) deleted or not fulfil the mode requirement. Too brief and not clear, kindly enhance.

Response: We have now added the removed pathways from the model 1 to obtain model 2 in the first paragraph of Structural Model subsection. Additional pathways based on the modification index recommendation were presented in the second paragraph.

e. Table 3 present the significant path, how about the non-significant path(s). The path deleted from the model during model fit or during the measurement model analysis or during the structural model analysis?

Response: We thank the reviewer for the comment. The path deleted from the model during the structural model analysis as stated in subsection Structural Model.

f. How the value of indirect effect calculates? Where the significant value from?

Response: The value of indirect effects and its p-values were obtained from the Mplus output. It is part of the SEM analysis. We have futher explained this in line 222.

3. What the age (years) of the participants? Kindly report in group, not mean.

Response: We thank the reviewer for the suggestion. We have now added age group of the participants in Table 1.

4. Discussion too brief and more focus on reported previous studies. Discussion should align with the purpose of the study.

a. What the overall contribution? -23% refer to good or moderate etc?

Response: The overall R^2^ on amount of PA indicated that 23% of variance in amount of PA could be explained by PA motives (others’ expectation and psychological condition) and decisional balance (Pros). We do not interpret the 23% as good or moderate, because the interpretation is very depend on the field of study. There is no gold standard available in the literature to label the level of coefficient of determination (R^2^). We report the values and allowed readers to make their own judgement.

b. What the contribution of indirect effect presented in this model outcome? What’s the value or impact of the findings?

Response: We have now added a paragraph in the discussion to explain on the value or impact of the findings on the participants. See line 389-400.

5. No implication and further research suggestion. Kindly revise and enchance the Discussion.

Response: We thank the reviewer for the suggetion. We have now added a paragraph (last) in the Discussion section to discuss future studies recommendations.

**Reviewer #2:**

1. Title: Include the place of study

Response:

We thank the reviewer for the suggestion. We have added place of study (Malaysia) in the title.

2. Abstract: the study design used was Mixed method, so the term cross sectional and purpose sampling are not important. sample size and sampling method for both quantitative and qualitative design need to be presented in the methods section.

Response: We thank the reviewer for the comment. We rephrased the sentence to indicate that was only a cross-sectional study design and non-probability sampling method purposive sampling was used. We also added the sample size of the present study in the method section.

3. Introduction: Psychologically, the transtheoretical model (TTM) is a questionnaire...(TTM is a behavior change model not a questionnaire).

Response: We have now rephrased the the sentence in the third paragraph.

4. Materials and Methods; the study design section needs clarity. sampling method and participant section or sampling procedure are not clear.describe each study designs used, how participants were chosen, how many etc. take the statement "All participants were initially required to complete consent forms before the study began as an indication that they volunteered and were willing to participate in this study" to ethical consideration section. the Measures and Materials is nice.from whom you collect the data? patient them selves or their medical card. some relevant variables such as religion are missed. if you used cards, it secondary data and you should clearly mention that you used secondary data and its limitations.

Response: We thank the reviewer for the comment. We added clarification in the first paragraph of Study design section regarding the cross-sectional study design and non-randomized sampling for this study. The inclusion and exclusion criteria of participants are included in the same paragraph. We also removed the statement "All participants were initially required to complete consent forms before the study began as an indication that they volunteered and were willing to participate in this study" to ethical consideration section. The data was collected from the patient themselves, not from their medical card. We did not collect their religion variables.

5. Results: the last two variables in table one are not demographic characteristics. Rather, they are clinical. the title of tables need to be written properly. what where and when for each table. the final model( the relationship between the pathways and the PA) should be interpreted in detail. This is the core of your research and same is true in discussion section. You use only a cross sectional quantitative study. at the beginning it seems you used a mixed method. In general it is a good work. there are some spelling errors like the word University in many sections. with careful revision, it will be a good article.

Response: We thank for the comment. The title of the tables have been revised properly. The final model has been further interpreted in detail in the Discussion section.
