# Supplementary material for: Structural equation model of psychological constructs of transtheoretical model, motives for physical activity, and amount of physical activity among people with type 2 diabetes mellitus in Malaysia
Source: PLoS One. 2022 Mar 31;17(3):e0266104. doi: 10.1371/journal.pone.0266104 (PMC8970478; doi:10.1371/journal.pone.0266104)
Supplement: Attachment — Submitted filename: Response to Reviewers.docx [file pone.0266104.s004.docx]

Dear Hoh Boon-Peng,

On behalf of my co-authors, I would like to thank you for your consideration of our manuscript, titled “Structural Equation Model of Psychological Constructs of Trantheoretical Model, Motives for Physical Activity, and Amount of Physical Activity among People with Type 2 Diabetes Mellitus in Malaysia”. We thank the Editor and the Reviewers for the careful consideration of our manuscript and for the largely favourable appraisal of this work. We also appreciate the Editor’s and Reviewers’ comments and think that the comments substantially helped us enhance the paper’s overall quality. As such, we highly appreciate the opportunity to revise and resubmit the paper. We hope that we have now adequately addressed the Editor’s and reviewer’ concerns and that you will now find our manuscript a useful addition to PLoS One. However, should there be any further issues requiring our attention, we would be grateful for a further opportunity to rectify these. Below, you will find our detailed responses to the comments.

**Editor’s comments and authors’ responses:**

**Reviewer 2:**

1. Thank you dear authors, i realize that most of the comments are corrected. However, i have still concern on the sample size determination and sample section. You need to show how you come up with 331 participants/calculate showing the formula and the parameters used and how you chose them. I am not convinced about the selection of your study participants. In addition, include how you collected the data and the quality control measure used .

Response: We thank you for the comment. We have added the sample size calculation using monte carlo simulation in Mplus software. New subsection for this part. Also, we added new subsection explaining on how data collection and participants’ recruitment were performed. References also revised according to the additional citation in the sample size calculation subsection.
